# NMR and Computational Studies as Analytical and High-Resolution Structural Tool for Complex Hydroperoxides and Diastereomeric *Endo*-Hydroperoxides of Fatty Acids in Solution-Exemplified by Methyl Linolenate

**DOI:** 10.3390/molecules25214902

**Published:** 2020-10-23

**Authors:** Raheel Ahmed, Panayiotis C. Varras, Michael G. Siskos, Hina Siddiqui, M. Iqbal Choudhary, Ioannis P. Gerothanassis

**Affiliations:** 1 H.E.J. Research Institute of Chemistry, International Center for Chemical and Biological Sciences, University of Karachi, Karachi 75270, Pakistan; genius88raheel@gmail.com (R.A.); iqbal.choudhary@iccs.edu (M.I.C.); 2 Section of Organic Chemistry and Biochemistry, Department of Chemistry, University of Ioannina, GR-45110 Ioannina, Greece; panostch@gmail.com (P.C.V.); msiskos@uoi.gr (M.G.S.); 3 Department of Biochemistry, Faculty of Science, King Abdulaziz University, Jeddah 214412, Saudi Arabia

**Keywords:** polyunsaturated fatty acids, hydroperoxides, *endo*-hydroperoxides, NMR, chemical shifts, hydrogen bonding, ^1^H-^13^C HMBC, DFT, 1D TOCSY, ONIOM

## Abstract

A combination of selective 1D Total Correlation Spectroscopy (TOCSY) and ^1^H-^13^C Heteronuclear Multiple Bond Correlation (HMBC) NMR techniques has been employed for the identification of methyl linolenate primary oxidation products without the need for laborious isolation of the individual compounds. Complex hydroperoxides and diastereomeric *endo*-hydroperoxides were identified and quantified. Strongly deshielded C–O–O–H ^1^H-NMR resonances of diastereomeric *endo*-hydroperoxides in the region of 8.8 to 9.6 ppm were shown to be due to intramolecular hydrogen bonding interactions of the hydroperoxide proton with an oxygen atom of the five-member *endo*-peroxide ring. These strongly deshielded resonances were utilized as a new method to derive, for the first time, three-dimensional structures with an assignment of pairs of diastereomers in solution with the combined use of ^1^H-NMR chemical shifts, Density Functional Theory (DFT), and Our N-layered Integrated molecular Orbital and molecular Mechanics (ONIOM) calculations.

## 1. Introduction

Polyunsaturated fatty acids (PUFAs) are a broad and diverse group of naturally occurring biomolecules with numerous physicochemical and biological properties, and diverse roles in health and nutrition [1,2,3]. PUFAs are particularly susceptible to oxidation upon exposure of thermal stress, light, photosensitizing pigments, and metal ions. This results in primary oxidation products, such as hydroperoxides, *endo*-hydroperoxides, hydroperoxide bis cyclic peroxides, and polymeric hydroperoxides. Primary oxidation products degrade into secondary oxidation products such as aldehydes, ketones, free acids, and hydroxyl compounds that in high levels can be harmful to human health. Lipid oxidation products have cytotoxic and genotoxic properties. They are implicated in the disruption of membranes, inactivation of enzymes and proteins, the formation of age pigments in broken membranes, oxidative harm to lungs through atmospheric pollutants, and cancers [1,2,3,4,5,6,7].

The general mechanism of oxidation is a free radical chain reaction that entails hydrogen abstraction at the allylic carbon with the formation of allylic radicals in which electrons are delocalized by 3-carbon (oleate) or 5-carbon (linoleate and linolenate) systems. The rearrangement of the double bonds results in the formation of conjugated dienes; attack by molecular oxygen produces a peroxy radical, which can abstract a hydrogen atom from an adjacent carbon [7,8,9]. This results in complex isomeric hydroperoxides. The oxidation of unsaturated fatty acids containing more than two double bonds, such as in linolenate, results in the formation of a mixture of hydroperoxides, *endo*-hydroperoxides (diperoxides), and hydroperoxy bis cyclic peroxides, along with secondary oxidation products [10,11,12].

Numerous analytical methods have been developed to detect fatty acid oxidation and its level, including traditional chemical assaying methods (peroxide value, thiobarbituric acid (TBA) test, anisidine value, etc.) [13], headspace GC-MS to measure secondary oxidation products, such as aldehydes and ketones [14,15], and HPLC PDA to investigate non-volatile oxidation products [16,17]. With the above analytical methods, the sample has to be processed, and the progress of oxidation should be interrupted. ^1^H- and ^13^C-NMR have also been extensively utilized to elucidate the structures of primary and secondary oxidation products both in model compounds and in edible oils, which were subjected to a variety of degradative conditions [18,19,20,21,22,23,24]. More recently, ^1^H-NMR of hydroperoxide (-OOH) chemical shifts and 1D band selective Nuclear Overhauser Enhancement Spectroscopy (NOESY) and TOCSY experiments were utilized for the specific assignments of various hydroperoxide positions [25].

We report herein an extension of our previous study on the use of ^1^H-^13^C HMBC NMR experiments as a structural and analytical tool for the identification and quantification of hydroperoxide isomers [26,27]. Complex hydroperoxides and *endo*-hydroperoxides of methyl linolenate were identified and quantified, with the combination of selective 1D TOCSY and ^1^H-^13^C HMBC NMR techniques without laborious isolation of the individual components. Their three-dimensional (3D) structures with assignment of pairs of diastereomers in solution were elucidated, for the first time, with the combined use of ^1^H-NMR chemical shifts, Density Functional Theory (DFT) and Our N-layered Integrated molecular Orbital and molecular Mechanics (ONIOM) calculations. 

## 2. Results and Discussion

### 2.1. NMR Studies (^1^H-^13^C HMBC and 1D TOCSY NMR)

**Figure 1:**
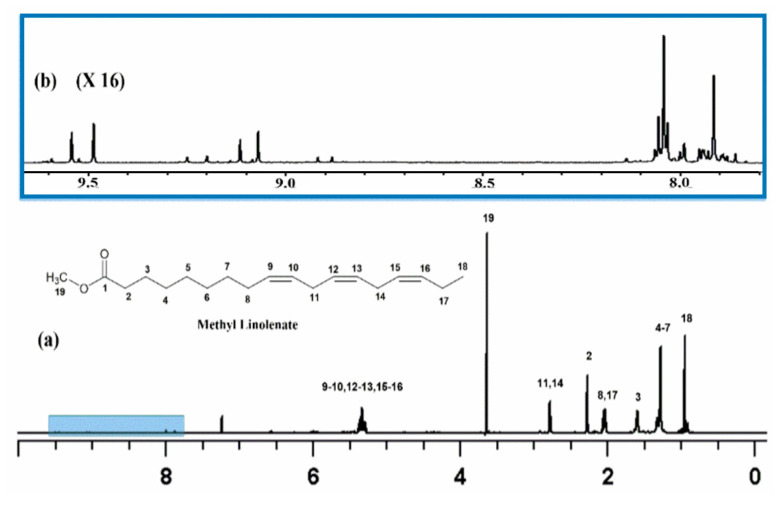
(**a**) 800 MHz ^1^H-NMR spectrum of 20 mg methyl linolenate in CDCl_3_ subjected to heating at 40 °C for 48 h, number of scans = 64, acquisition time = 1.02 s, experimental time = 6.5 min, relaxation delay = 5 s, T = 298 K; (**b**) selected region of the C–O–O–H resonances of its primary oxidation products.

**Figure 2:**
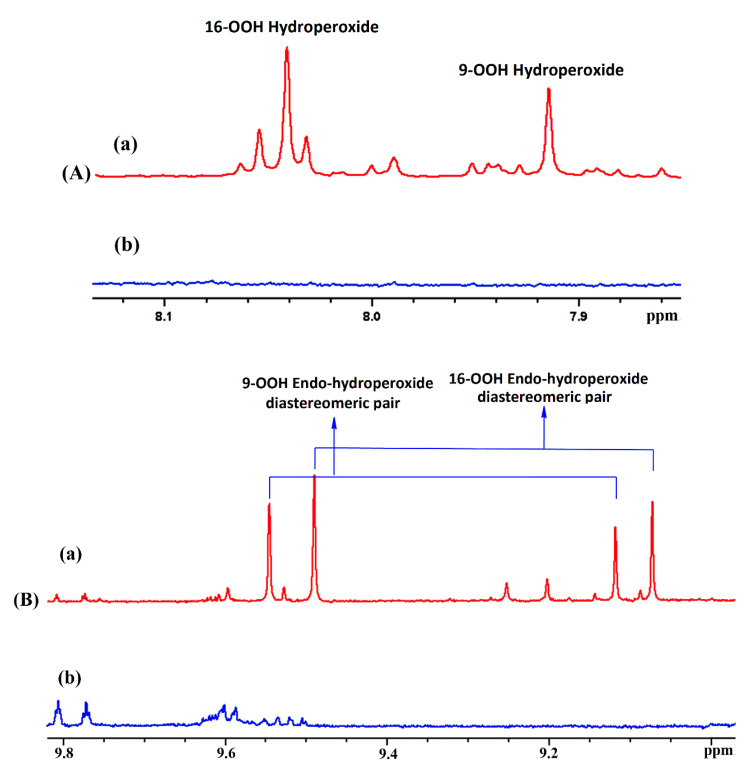
800 MHz ^1^H-NMR spectra of the solution of Figure 1 before the addition (**a**), and after the addition (**b**) of 2 microdrops of D_2_O. (**A**,**B**) are the selected regions of the 7.86 to 8.14 and 8.96 to 9.82 ppm, respectively. For the method of assignment of the -OOH resonances, see text.

Methyl linolenate was selected as a suitable representative of omega-3 fatty acids that contains three methylene intruded *cis* double bonds. Figure 1 illustrates the ^1^H-NMR spectrum of a sample of methyl linolenate, which was allowed to oxidize in atmospheric oxygen in a glass vial for 48 h in a conventional oven at 40 °C, and it was subsequently dissolved (20 mg of sample in CDCl_3_). A very significant number of sharp ^1^H-NMR resonances (Δν_1/2_ ≤ 2.0 Hz) of the hydroperoxide C–O–O–H protons in the region of 7.7 to 9.6 ppm was observed. The addition of 1 to 2 microdrops of D_2_O resulted in the elimination of the majority of these signals including those in the strongly deshielded region of 8.8 to 9.6 ppm (Figure 2), where resonances of saturated and unsaturated aldehydes, as secondary oxidation products, would be expected to appear.

Optimization of the ^1^H–^13^C HMBC experiments for an effective magnetization transfer from the hydroperoxide proton to the methine CH–O–O–H carbon would require the knowledge of the ^3^*J*(C–O–O–H) coupling constant which strongly depends upon the C–O–O–H torsion angle φ_1_. For hydroperoxides, a wide range of φ_1_ angles (100° ± 20°) were found crystallographically [28], which correspond to the minimum size of the ^3^*J* couplings in the Karplus equation. Furthermore, since the presence of electronegative atoms is expected to reduce the value of the ^3^*J* coupling constants, the ^1^H–^13^C HMBC experiment was optimized for an average ^3^*J*(C–O–O–H) coupling of ≈4 Hz.

**Figure 3:**
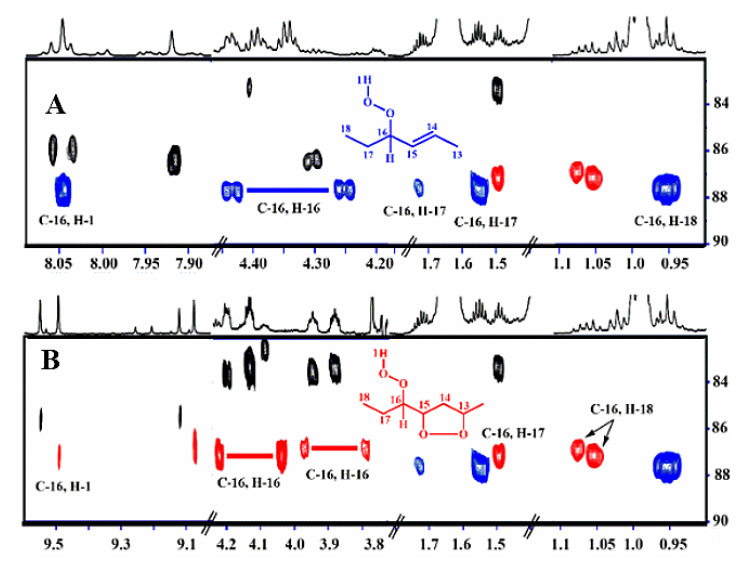
Selected regions of 800 MHz ^1^H-^13^C HMBC NMR spectrum of 20 mg methyl linolenate in CDCl_3_, subjected to heating at 40 °C for 48 h, number of scans = 32, number of increments 256, total experimental time = 12 h, T = 298 K. The critical cross-peak connectivities of C-16 with H-1(OOH), H-16 (^1^*J*(^13^C, ^1^H)), H-17, and H-18 of the 9-*cis*, 12-*cis,* 14-*trans*-16-OOH hydroperoxide (**A**), and two diastereomeric 9-*cis*, 11-*trans*-16-OOH *endo*-hydroperoxides (in red) (**B**), are illustrated.

**Figure 4:**
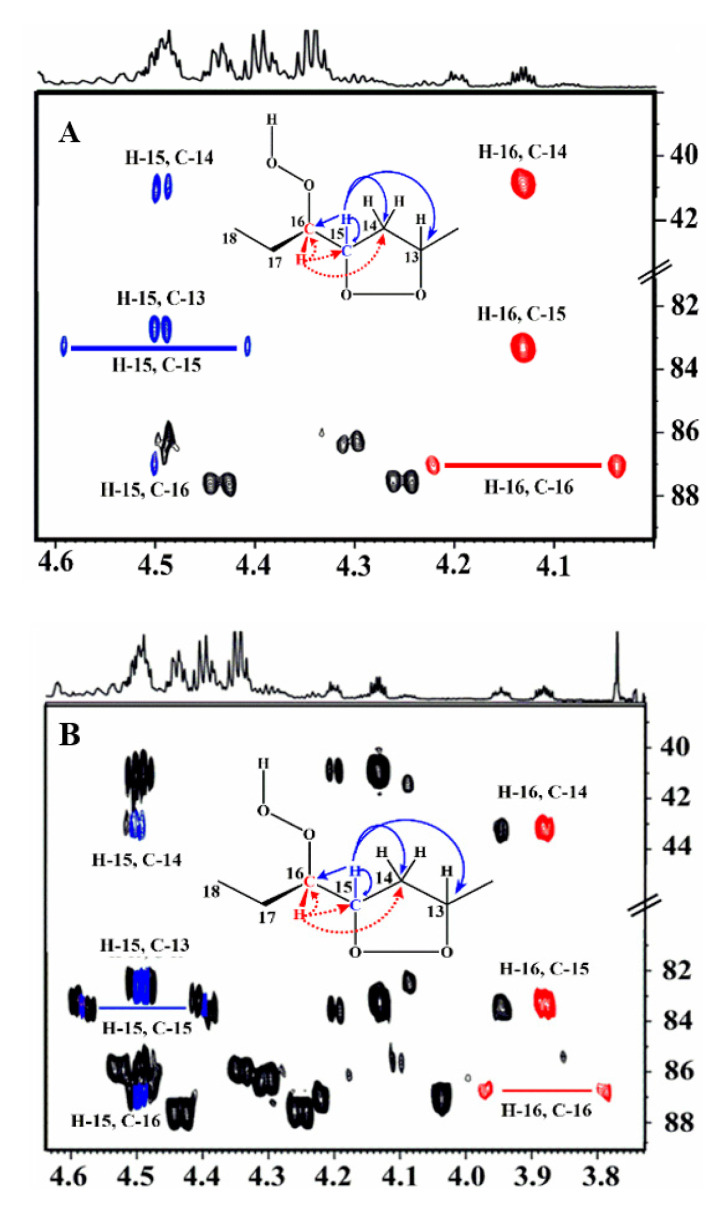
^1^H-^13^C HMBC correlations showing the assignments of the two diastereomeric 9-*cis*, 11-*trans*-16-OOH *endo*-hydroperoxides of methyl linolenate with OOH and CH-OOH resonances at 9.50 ppm and 4.3 ppm, respectively (**A**), and 9.08 ppm and 3.87 ppm, respectively (**B**). The critical connectivities of the H-15 with C-14, C-13, C-15 (^1^*J*(^13^C, ^1^H)), C-16, and connectivities of H-16 with C-14, C-15, and C-16 (^1^*J*(^13^C, ^1^H)) are illustrated.

Figure 3 illustrates a selected region of the ^1^H-^13^C HMBC spectrum. Despite the low concentration of the hydroperoxides and *endo*-hydroperoxides (≈1.36–8.23%), the ^1^H-^13^C HMBC experiment revealed several ^13^C connectivities between *δ* 82 and 88 for the C–O–O–H carbons. Furthermore, the ^1^H-^13^C HMBC spectrum demonstrates very informative connectivities of the C–O–O–H carbon at *δ* of 87.5, with the terminal –CH_3_ (*δ* 0.9) and CH_3_–CH_2_ (*δ* 1.72, 1.55) protons, thus confirming the presence of the 16–OOH hydroperoxide. Of diagnostic importance are also the ^1^H-^13^C HMBC connectivities of the two deshielded terminal CH_3_ groups at *δ* 1.05 and 1.07 with the respective hydroperoxide carbons (*δ* 87.2, and 87.0) and C–O–O–H protons in the strongly deshielded region at *δ* 9.50 and 9.08, respectively (Figure 3). Furthermore, their CH–O–O–H protons at *δ* 4.13 and 3.87 demonstrate very informative additional connectivities in the region of the hydroperoxide carbons (Figure 4). This supported an unequivocal presence of two 16–OOH *endo*-hydroperoxide isomers of the oxidized fatty acid sample. Thus, the H-16 multiplet at *δ* 4.13 demonstrates connectivities with C-16 (*δ* 87.2), C-15 (*δ* 83.35), and C-14 (*δ* 40.7) (Figure 4A). The C-16 also shows connectivities with the H-15 multiplet (*δ* 4.49), which shows a characteristic doublet with C-15, due to ^1^*J*(^13^C,^1^H) of 146 Hz, and connectivities with C-13 (*δ* 82.8) and C-14. Similar connectivities were observed for the H-16 multiplet (*δ* 3.87) of the second diastereoisomer (Figure 4B). 

**Figure 5:**
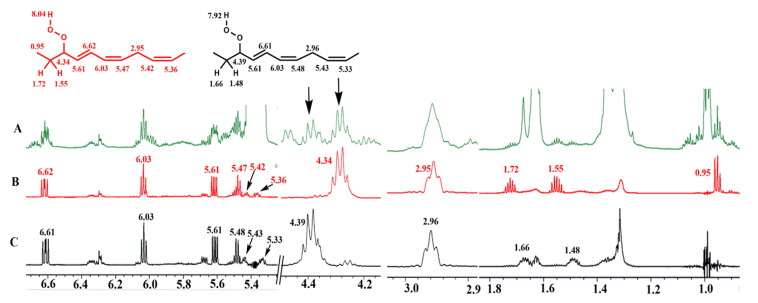
(**A**) 800 MHz 1D ^1^H-NMR spectrum of 20 mg methyl linolenate, subjected to heating at 40 °C for 48 h, in CDCl_3_ (acquisition time= 1.02 s, relaxation delay = 5 s, number of scans = 128, experimental time = 10 min), T = 298 K. (**B**,**C**) selective 1D TOCSY spectra of the same solution using a mixing time of τ_m_ = 300 ms. The arrows denote the selected resonances that were excited at *δ* 4.34 (**B**) of the 9-*cis*, 12-*cis*, 14-*trans*-16-OOH hydroperoxide and *δ* 4.39 (**C**) of the 10-*trans*, 12-*cis*, 15-*cis*-9-OOH hydroperoxide. Number of scans = 256, experimental time = 20 min.

Furthermore, the identification of the above hydroperoxides was confirmed with the use of selective 1D TOCSY experiments [29]. An important application of the 1D experiment is the selective excitation of a suitable target resonance of the compound of interest, which reveals an extensive spin system belonging to a single chemical analyte, even in heavily overlapped resonances [30,31,32,33,34]. Selective excitation of the allylic CH–O–O–H proton of the 9-*cis*, 12-*cis*, 14-*trans*-16-OOH geometric hydroperoxide isomer at *δ* 4.34 (Figure 5B) shows connectivities with the terminal C-18 methyl group at *δ* 0.95, the H-17 (*δ* 1.72 and 1.55), the conjugated olefinic protons H-15 (*δ* 5.61), H-14 (*δ* 6.62), H-13 (*δ* 6.03), H-12 (*δ* 5.47), the H-11 bis allylic protons (*δ* 2.95), the olefinic protons H-10 (*δ* 5.42), and H-9 (*δ* 5.36). The selective excitation of the 10-*trans,* 12-*cis*, 15-*cis*-9-OOH isomer at *δ* 4.39 shows connectivities with H-8 (*δ* 1.66 and 1.48), the conjugated olefinic protons H-10 (*δ* 5.61), H-11 (*δ* 6.61), H-12 (*δ* 6.03), H-13 (*δ* 5.47), the H-14 bis allylic protons (*δ* 2.96), the olefinic H-15 (*δ* 5.43), and H-16 (*δ* 5.33) (Figure 5C).

**Figure 6:**
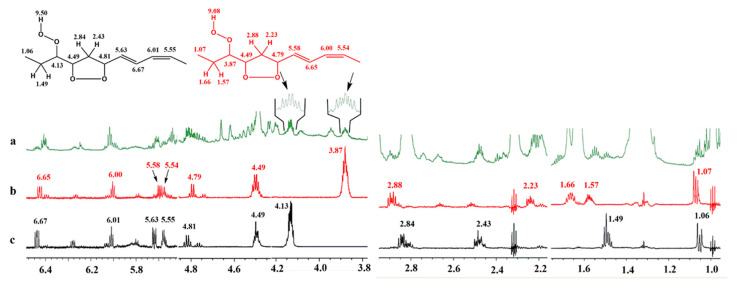
(**a**) 800 MHz 1D ^1^H-NMR spectrum of 20 mg of methyl linolenate, subjected to heating at 40 °C for 48 h, in CDCl_3_ (T = 298 K, acquisition time = 1.02 s, relaxation delay = 5 s, number of scans = 128, experimental time = 10 min). (**b**,**c**) selective 1D TOCSY spectra of the same solution using a mixing time of τ_m_ = 300 ms. The arrows denote the selected resonances of the two diastereomeric 9-*cis*, 11-*trans*-16-OOH *endo*-hydroperoxides that were excited at *δ* 3.87 (**b**) and *δ* 4.13 (**c**).

The identification of the two diastereomeric 9-*cis*, 11-*trans*-16-OOH *endo*-hydroperoxides can be achieved by the selective excitation of the CH–O–O–H allylic protons at *δ* 4.13 and 3.87. Thus, selective excitation of the multiplet at *δ* 3.87 (Figure 6b) results in connectivities with the H-17 (*δ* 1.66 and 1.57), the terminal CH_3_ group (*δ* 1.07), H-15 (*δ* 4.40), H-14 (*δ* 2.88 and 2.23), H-13 (*δ* 4.79), and with the conjugated double bond protons of H-12 (*δ* 5.58), H-11 (*δ* 6.65), H10 (*δ* 6.00), and H-9 (*δ* 5.54). Similar long-range connectivities were observed with the selective excitation of the multiplet at *δ* 4.13 (Figure 6c).

**Figure 7:**
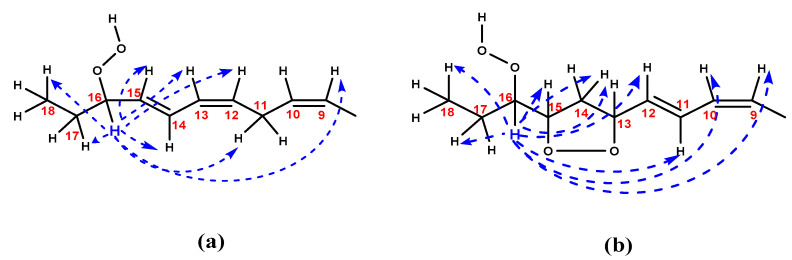
Schematic presentation of the selective 1D TOCSY correlations which were observed in the case of the 9-*cis*, 12-*cis*, 14-*trans*-16-OOH hydroperoxide (**a**), and the 9-*cis*, 11-*trans*-16-OOH *endo*-hydroperoxide (**b**) of the methyl linolenate primary oxidation products.

Figure 7 illustrates schematically the unique advantages of the selective 1D TOCSY experiment. The method allows, through a careful selection of the spin system to be excited, the unambiguous identification of an extensive spin coupled system of minor abundance hydroperoxides and *endo*-hydroperoxides even in the cases of strongly overlapping resonances.

Similar experiments were performed for the strongly deshielded hydroperoxide protons at 9.55 and 9.12 ppm (Figure 2B), which were assigned to the two diastereomeric 13-*trans*, 15-*cis*-9-OOH *endo*-hydroperoxides. Of particular interest are the significant differences of the OOH chemical shifts of the two diastereomeric pairs of 9-*cis*, 11-*trans*-16-OOH, and 13-*trans*, 15-*cis*-9-OOH *endo*-hydroperoxides which allowed, through ^1^H-^13^C HMBC experiments, the identification of the respective CH-OOH protons that also have very significant chemical shift differences (Table S1). Figure S1A illustrates the ^1^H NMR chemical shift range of the *endo*-hydroperoxides, which shows sufficient resolution even when using a 400 MHz instrument. However, the proposed methodology implies ^1^H-^13^C HMBC connectivities of the -OOH with the CH-OOH protons. The achievable resolution of the CH-OOH protons using a 400 MHz instrument is not sufficient for identification due to strong signal overlapping (Figure S1B). However, the ^1^H-NMR spectrum using a 600 MHz instrument is nearly identical to that obtained at 800 MHz, which demonstrates that the proposed experiments can be performed using a 600 MHz instrument.

**Figure 8:**
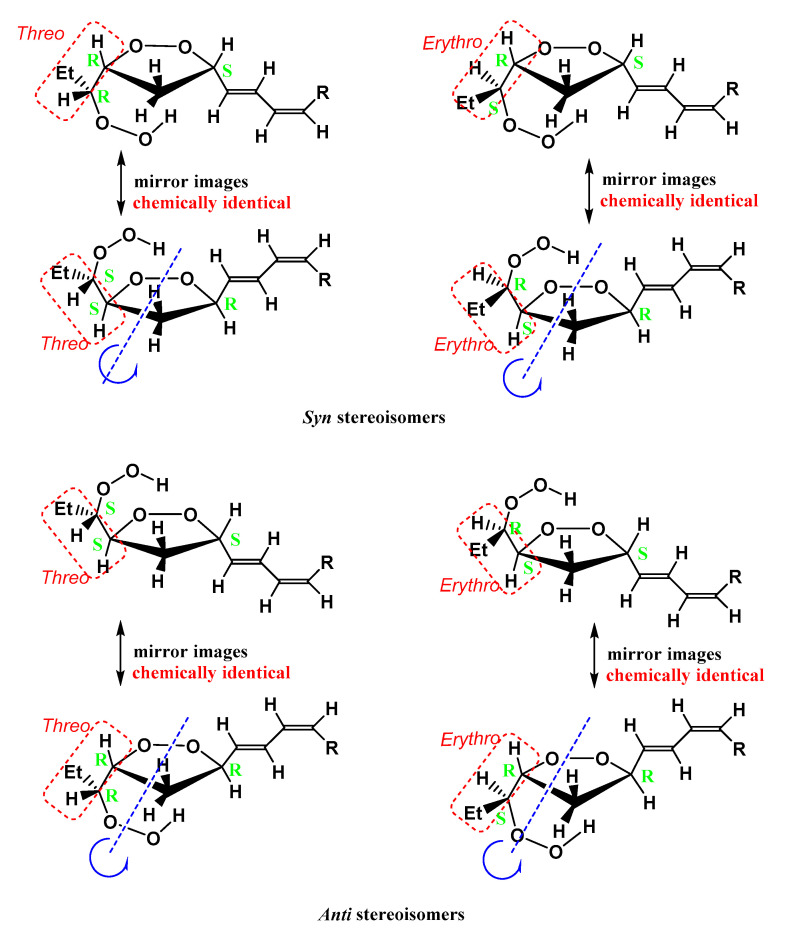
The 2^3^ = 8 stereoisomers (2^3–1^ = 4 pairs of enantiomers) of 9-*cis*, 11-*trans*, 16-OOH linolenate *endo*-hydroperoxides investigated in the present work.

The formation of two pairs of 9-*cis*, 11-*trans*-16-OOH and 13-*trans*,15-*cis*-9-OOH *endo*-hydroperoxides of methyl linolenate has been attributed to hydrogen abstraction at carbons 11 and 14 (Figure S2), which results in the formation of two pentadienyl radicals. Reaction with oxygen at the end carbon positions produces a mixture of four peroxyl radicals leading to the corresponding conjugated dienoic 9-, 12-, 13-, and 16-hydroperoxides [10,12,35,36,37,38]. The peroxyl radicals derived from internal 12- and 13-hydroperoxides undergo rapid 1,5-cyclization to form two five-membered isomeric 9- and 16-hydroperoxy cyclo-*endo*-peroxides (Figure S2). In the case of e.g., 13-OOH hydroperoxide, the presence of three stereocenters C-13, C-15, and C-16 imply a number of 2^3^ = 8 stereoisomers (2^3–1^ = 4 pairs of enantiomers) of 9-*cis*, 11-*trans*-16-OOH linolenate *endo*-hydroperoxides (similar arguments also imply in the case of 13-*trans*, 15-*cis*-9-OOH hydroperoxides) (Figure 8). Only one pair of 16-OOH/9-OOH *endo*-hydroperoxides was identified by the use of preparative high-pressure liquid chromatography (HPLC) [4,35]. This aspect and the correct stereoisomerism of the resulting *endo*-hydroperoxides were investigated in detail with the use of DFT calculations (see discussion below). 

### 2.2. Quantum Chemical Calculations 

The development of quantum chemical methods for calculating NMR chemical shifts has led to a significant number of studies that combine experimental chemical shifts with computations [39,40,41,42]. Nevertheless, a few examples of organic molecules whose structures have been determined by both computations of NMR chemical shifts in solution and X-ray structures have been published [43,44,45,46,47,48,49]. Since no X-ray structures of the hydroperoxides of fatty acids have so far been reported, it would be of interest to investigate three-dimensional structures of hydroperoxides of the present work, which are based on the combined use of quantum chemical calculations and ^1^H-NMR chemical shifts.

**Figure 9:**
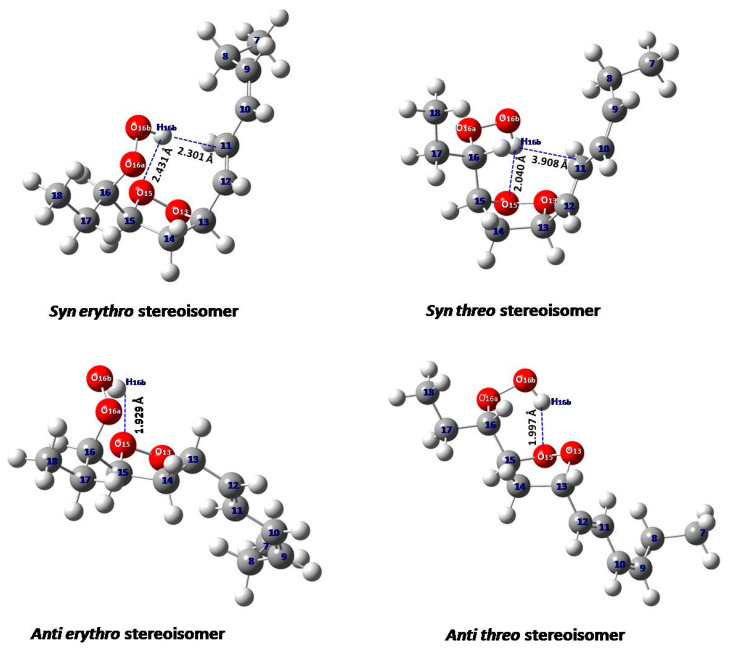
Minimum energy structures of two pairs of diastereomers of 9-*cis*, 11-*trans*-16-OOH *endo*-hydroperoxide models in integral equation formalism polarizable continuum model (IEFPCM) (CHCl_3_).

Firstly, we investigated models of the two pairs of diastereoisomeric *9*-*cis, 11-trans*-16-OOH *endo*-hydroperoxides (carbons 18 to 7) (Figure 9) and a model of 9-*cis*, 12-*cis,* 14-*trans*-16-OOH hydroperoxide (carbons 18 to 9) using the DFT quantum chemical method [50,51]. All optimizations were performed using the Austin-Frisch-Petersson functional with Dispersion (APFD) along with the 6-31+G(d) basis set: (i) in the gas phase, (ii) using the integral equation formalism polarizable continuum model (IEFPCM) in CHCl_3_, and (iii) with a discrete solvation molecule of CHCl_3_. Vibrational analysis was used to verify the nature of the stationary points. Secondly, we have also used the Our N-layered Integrated molecular Orbital and molecular Mechanics (ONIOM) method [52] to study the full-length compounds and compare the results with the model compounds. Furthermore, the NMR calculations were carried out at the optimized geometries, which were obtained with the DFT and ONIOM methods, using the B3LYP functional and the 6-311+G(2d,p) basis set. For comparison purposes, the NMR calculations were performed using the GIAO (Gauge-Independent Atomic Orbital) and the CSGT (Continuous Set of Gauge Transformations) methods [53].

#### 2.2.1. DFT Studies of Model Compounds—Assigning the Stereochemistry of Pairs of Diastereoisomers

The minimum energy DFT structures of the two pairs of diastereoisomers of 9-*cis*, 11-*trans*-16-OOH *endo*-hydroperoxides are illustrated in Figure 9. Table 1 shows that the conformational and structural properties of the four stereoisomers remain essentially the same in the gas phase, in IEFPCM (CHCl_3_) and with a discrete solvation molecule of CHCl_3_ (Figure S3). The *syn threo* stereoisomer shows a hydrogen bond interaction of the hydroperoxide proton, H16_b_, with the O_15_ of the five-member *endo*-peroxide ring. The distance H16_b_^…^O15 of 2.040 Å indicates the presence of a medium-strength hydrogen bond interaction. In the *syn erythro* stereoisomer, the interaction of the hydroperoxide proton H16_b_ with the π-electrons of the conjugated double bond results in a very weak interaction with the oxygen O_15_ of the five-member *endo*-peroxide ring (H16_b_^…^O15 = 2.431 Å), which is in the limits of the definition of hydrogen bond interaction (Figure 9). 

The *anti erythro* and *anti threo* stereoisomers, with the -OOH and conjugated substituents in *anti*-position, show the hydrogen bond interaction of the hydroperoxide proton H16_b_ with the O15 of the five-member *endo*-peroxide ring. The distances H16_b_^…^O15 of 1.929 Å and 1.997 Å indicate the presence of stronger hydrogen bond interaction than in the *syn threo* and, especially, in *syn erythro* stereoisomer. However, in the region of strongly deshielded hydroperoxide protons (9.0 to 9.8 ppm, Figure 2B), only four prominent resonances were observed. These resonance were unequivocally assigned, with the combined use of ^1^H-^13^C HMBC and selected 1D-TOCSY experiments (see Section 2.1), to a diastereomeric pair of 9-*cis*, 11-*trans*-16-OOH *endo*-hydroperoxide (*δ* = 9.50 and 9.08 ppm) and a diastereomeric pair of 13-*trans*, 15-*cis*-9-OOH *endo*-hydroperoxide (*δ* = 9.55 and 9.12 ppm) (Figure 2B). Similarly, only two diastereomeric pairs were isolated with the use of preparative high-pressure liquid chromatography (HPLC) [4,35].

Table S2 shows a comparison of computational ^1^H-NMR chemical shifts of the four model diastereomeric *endo*-hydroperoxides with the experimental chemical shifts of the full-length molecules. The *syn threo* stereoisomer, both in PCM and with the inclusion of a discrete solvation molecule of CHCl_3_ in PCM, shows a strong magnetic non-equivalence of the C(14)Ha,b protons, which is in excellent agreement with the experimental data. On the contrary, the calculated C(14)Ha,b chemical shifts are very similar in the *syn threo* stereoisomer. This supports the conclusion that the stereoisomer with *δ*(OOH) = 9.50 ppm can be assigned to 9-*cis*, 11-*trans*, *syn threo*, 16-OOH *endo*-hydroperoxide. The *syn erythro* and *anti erythro* stereoisomers show strong magnetic non-equivalence of the C(14)Ha,b protons in both PCM and with the inclusion of a discrete solvation molecule of CHCl_3_ (Table S2); thus, they cannot be distinguished on the basis of this criterion. However, the *syn erythro* stereoisomer shows very weak interaction of the hydroperoxide proton with the O15 of the five-member *endo*-peroxide ring, contrary to the medium hydrogen bond interaction of the *anti erythro* stereoisomer (Figure 9 and Figure S2). Therefore, the resonance at 9.08 ppm, which is shielded relative to that at 9.50 ppm, may be assigned to 9-*cis*, 11-*trans*, *syn erythro*, 16-OOH *endo*-hydroperoxide.

Table S3 shows a comparison of experimental and computational ^1^H-NMR chemical shifts of the 10-*cis*, 13-*cis,* 15-*trans*-16-OOH hydroperoxide model with the chemical shifts of the full-length molecule. The inclusion of one solvation molecule of CHCl_3_ improves the agreement between computational and experimental ^1^H-NMR chemical shifts of the solvent-exposed hydroperoxide proton. 

#### 2.2.2. DFT and ONIOM Computational Studies of the Full Length 16-OOH Endo-Hydroperoxides

**Figure 10:**
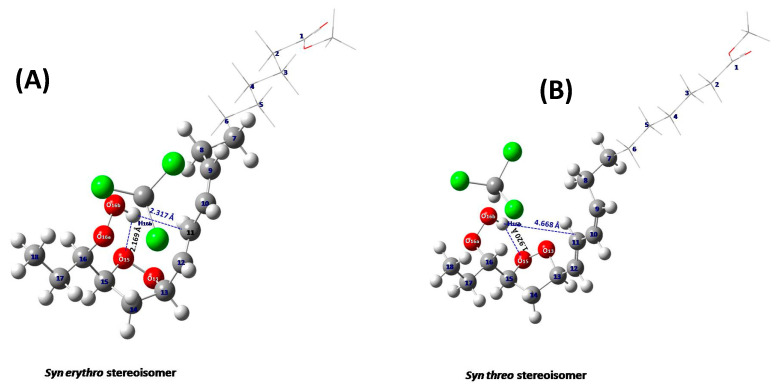
Structural comparison of the full-length *syn erythro* (**A**) and *syn threo* (**B**) *endo*-hydroperoxides, with a discrete solvation molecule of CHCl_3_ (energy minimization using the APFD/6-31+G(d):PM6 method).

According to the ONIOM method, the molecule under study is divided into two layers, the high and the low layer, where the most important part of the molecule is placed in the high layer and treated with an accurate quantum chemical model such as the DFT method. The less important part is placed in the low layer and treated with a less expensive method, such as a semi-empirical (PM6). Figure 10 illustrates the structural comparison of the full-length *syn erythro*- (A) and *syn threo*- (B) *endo*-hydroperoxides with energy minimization using the APFD/6-31+G(d):PM6 method. Table S4 shows a comparison of experimental and computational ^1^H-NMR chemical shifts of the full-length (carbons 1 to 18) diastereomeric 9-*cis*, 11-*trans*-16-OOH *endo*-hydroperoxides with energy minimization at the APFD/6-31+G(d): PM6 level. The chemical shifts, structural, and conformational properties of the C–O–O–H hydroperoxide unit and the five-membered epidioxide ring are very similar to those of the model compounds of Figure S3 and Table S2. The computed and experimental ^1^H-NMR chemical shifts of the hydroperoxide proton are in agreement with a stronger hydrogen bond interaction of the hydroperoxide proton with the O15 oxygen of the five-member *endo*-peroxide ring in the major 15,16-*threo* than in the 15,16-*erythro endo*-hydroperoxide molecule (Figure 10, Table S5) in excellent agreement with the structural data of the model compounds (Figure 9, Table 1). 

A large number of hydrogen bonds in crystal structures of alkyl hydroperoxides have been reported [28]. For some compounds, the formation of an intramolecular hydrogen bond would in principle have been feasible. Nevertheless, the number of examples in the literature so far is in favor of intermolecular rather than intramolecular hydrogen bonds. Therefore, no direct comparison with the structural data of Table S5 could be made. 

### 2.3. Comparison with Literature Data

Table 2 presents a comparison of the ^1^H- and ^13^C-NMR experimental chemical shifts of *endo*-hydroperoxides of methyl linolenate of the present work with those reported in the literature for HPLC-isolated *endo*-hydroperoxides. The deviation of *δ* (^1^H) was found to be ≤0.02 ppm with the exception of the chemical shift of the hydroperoxide proton, which is expected to be very sensitive to inter- and intramolecular hydrogen bonds, temperature, and solution conditions. The deviation of *δ*(^13^C) was found to be ≤0.6 ppm. Therefore, it can be concluded that the agreement of the present NMR data with those of isolated analytes is excellent. 

Table S6 presents the ^1^H NMR chemical shifts of 9-*cis,* 12-*cis,* 14-*trans*-16-OOH and 10-*trans,* 12-*cis,* 15-*cis*-9-OOH hydroperoxides of methyl linolenate. However, to the best of our knowledge, there are no literature data of ^1^H-NMR chemical shifts for comparison. 

The excellent resolution achievable in the region of hydroperoxide C–O–O–H protons (*δ* 7.70 to 9.6, Figure 1 and Figure 2) allowed an accurate integration and, thus, quantification of primary oxidation products. Table 3 shows quantification of the hydroperoxide ^1^H-NMR resonances based on their integration, with respect to the total integral of the CH_3_- group at 0.86 ppm. A comparison with literature integration data [36] shows very good agreement, despite differences in experimental conditions. As pointed out [10,12,35,36,37,38], the peroxyl radicals derived from 12- and 13- hydroperoxides undergo rapid 1,5-cyclization to form two five-membered isomeric internal 9- and 16-hydroperoxy cyclo *endo*-peroxides. This rapid cyclization results in lower concentrations of the internal 12- and 13-hydroperoxides (14% and 15%, respectively) relative to the external 9- and 16- hydroperoxides (29% and 41%, respectively) (Table 3). Similar relative concentrations of hydroperoxides have been found in the literature under a wide range of oxidation conditions [10,12,35,36,37,38]. 

## 3. Materials and Methods

### 3.1. Materials

Methyl linolenate (methyl (9Z,12Z,15Z)-octadeca-9,12,15-trienoate) and deuterochloroform (CDCl_3_, 99.8 d-%,) were purchased from Sigma-Aldrich (St. Louis, MO, USA). 

### 3.2. Oxidation Methodology

Methyl linolenate (200 mg) was oxidized in a glass vial (10 mL) at 40 °C for 48 h in the conventional oven (Labtech Windsor, Australia) circulating the atmospheric oxygen. 

### 3.3. NMR Methodology

^1^H-NMR experiments were recorded at 298 K on a Bruker AVANCE III HD 800 spectrometer with 128 scans and a relaxation delay of 5 s. One-dimensional TOCSY experiments were recorded at 298 K on a Bruker AVANCE III HD 800 spectrometer, using the MLEV-17 (selmlgp) pulse program, 128 scans, and a relaxation delay of 5 s. Selective excitation, with a bandwidth of 12 Hz (≈0.015 ppm), was achieved with a gradient-based echo block that uses a soft Gaussian 180° selective refocusing pulse of 49 ms, with power attenuation as indicated in Figure S4. The mixing time for long-range connectivities was set in the range of 200 to 300 ms. ^1^H–^13^C HMBC experiments were recorded at 298 K on a Bruker AVANCE III HD 800 spectrometer. The magnitude mode gradient enhanced HMBC experiment (hmbcgndqf pulse program) was acquired in the quadrature mode, with data points set to 4 k × 256 (t2 × t1) (total experimental time 12 h) or 4 k × 512 (t2 × t1) (total experimental time 24 h), with 4 k × 1 k (t2 × t1) data points for transformation, relaxation delay of 5 s, number of scans 32, with a sine function for apodization. The long-range coupling time was set to 125 ms (optimized for ^n^*J*_CH_ ≈4 Hz), SW = 10,416.66 Hz (F1) and 46,285.23 Hz (F2). 

### 3.4. DFT and ONIOM Calculations

The DFT computational study was performed by using the Gaussian 09 [54]. The structures were optimized by using the APFD functional and the 6-31+G(d) basis set in the gas phase, using the IEFPCM model in CHCl_3_, and with the inclusion of a discrete solvation molecule of CHCl_3_. The ^1^H-NMR chemical shifts were calculated with the GIAO and CSGT methods by using the B3LYP/6-311+G (2d, p) level with the PCM (polarizable continuum model) [55]. The optimized geometries were verified by performing frequency calculations at the same level (zero imaginary frequencies). TMS was used as a reference for the computed ^1^H-NMR chemical shift, and the optimization of TMS was calculated at the same level. The ONIOM optimizations were performed at the ONIOM(APFD/6-31+G(d):PM6) level of theory.

## 4. Conclusions

In this work, we have demonstrated that the combined use of 1D selective TOCSY and ^1^H-^13^C HMBC NMR techniques, DFT, and ONIOM computational studies is a powerful approach for obtaining detailed analytical and high-resolution structural information of hydroperoxides and diastereomeric *endo*-hydroperoxides in *ω*-3 fatty acid substrates. The proposed approach is of primary importance in hydroperoxide research because: 

(a) It is rapid, selective, and does not require derivatization steps.

(b) It allows the unequivocal identification and quantification of minor analytes even in strongly overlapped spectral regions, and thus, it is of importance in the emerging field of lipidomics [56,57,58,59,60,61], provided that a spectrometer with ^1^H resonance frequency ≥600 MHz is used.

(c) It can provide an excellent method for obtaining three-dimensional structures with diastereomeric assignment in solution [62]. 

Further NMR and computational studies to establish the identities of hydroperoxide-OOH resonances are currently in progress with *cis*-5,8,11,14,17-eicosapentaenoic acid (EPA) and *cis*-4,7,10,13,16,19-docosahexaenoic acid (DHA) methyl esters. 

## Figures and Tables

**Table 1:** Conformational and structural properties of two pairs of diastereomers of the model 9-*cis*, 11-*trans*-16-OOH *endo*-hydroperoxides with energy minimization using the APFD/6-31+G(d) method in the gas phase and with a discrete solvation molecule of CHCl_3_.

Compound	C–O (Å)	O–O (Å)	O–H (Å)	C(16) –O–O–H (°)	C(17)–C(16)–O–O (°)	C(15)–(16) –O–O (°)	(O)H^…^O (Å)	O(H)^…^O (Å)	O–H^…^O (°)	O(H)^…^C11 (Å)
*Syn erythro*	1.422	1.428	0.978	−90.0	−153.3	85.0	2.431	2.882	107.7	2.301
*Syn erythro (PCM)*	1.425	1.430	0.979	−88.9	−155.5	83.0	2.405	2.862	107.9	2.275
*Syn threo*	1.422	1.431	0.976	78.2	157.1	−82.4	2.039	2.761	129.0	3.910
*Syn threo (PCM)*	1.427	1.433	0.978	73.9	157.4	−81.9	1.990	2.743	132.1°	4.078
*Anti erythro*	1.422	1.432	0.979	−69.0	−154.9	83.3	1.981	2.730	131.4	
*Anti erythro (PCM)*	1.426	1.434	0.980	−66.7	−156.1	82.1	1.950	2.715	133.1	
*Anti threo*	1.422	1.431	0.977	73.2	155.3	−83.8	1.997	2.752	132.3	
*Anti threo (PCM)*	1.427	1.434	0.979	71.1	157.1	−82.1	1.944	2.724	134.8	
*Syn erythro + CHCl_3_*	1.424	1.430	0.979	−79.6	−153.4	84.6	2.184	2.800	119.5	2.307
*Syn erythro + CHCl_3_ (PCM)*	1.426	1.431	0.979	−78.9	−154.3	83.8	2.169	2.791	120.0	2.317
*Syn threo + CHCl_3_*	1.425	1.431	0.979	70.8	158.4	−80.8	1.948	2.719	133.8	4.591
*Syn threo + CHCl_3_ (PCM)*	1.429	1.433	0.980	68.9	158.6	−80.3	1.921	2.707	135.3	4.664
*Anti erythro + CHCl_3_*	1.426	1.433	0.981	−69.0	−154.5	83.3	1.928	2.721	136.0	
*Anti erythro + CHCl_3_ (PCM)*	1.430	1.433	0.982	−62.8	−154.7	82.9	1.920	2.716	136.3	
*Anti threo + CHCl_3_*	1.421	1.434	0.978	79.6	159.4	−79.5	1.982	2.692	127.5	
*Anti threo + CHCl_3_ (PCM)*	1.426	1.434	0.979	77.4	159.9	−79.1	1.958	2.685	129.1	

**Table 2:**
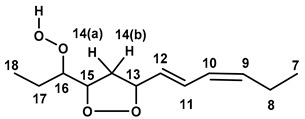
Comparison of experimental ^1^H- and ^13^C-NMR chemical shifts of the 9-*cis*, 11-*trans*-16-OOH diastereomeric *endo*-hydroperoxides of methyl linolenate of the present work and with the reported literature data with the use of isolated *endo*-hydroperoxides.

	9-*cis*, 11-*trans*-16-OOH *endo*-peroxy (threo)			9-*cis*, 11-*trans*-16-OOH *endo*-peroxy (erythro)		9-*cis*, 11-*trans*-16-OOH *endo*-peroxy (threo)	
**Proton no.**	***δ* (^1^H),** **ppm**	***δ* (^1^H),** **ppm ^a^**	***δ* (^1^H),** **ppm ^b^**	***δ* (^1^H),** **ppm**	***δ* (^1^H),** **ppm ^a^**	***δ* (^13^C),** **ppm**	***δ* (^13^C),** **ppm ^b^**
18	1.05	1.04	1.03	1.07	1.05	10.0	10.2
17	1.49	---	1.89−1.20	1.66	---	22.2	22.8
16	4.13	4.14	4.15	3.87	3.86	87.2	87.4
15	4.49	4.49	4.49	4.49	4.48	83.3	83.5
14(a)	2.84	2.86	2.84	2.88	2.88	40.7	41.3
14(b)	2.48	2.46	2.47	2.23	2.23		
13	4.81	4.80	4.80	4.79	4.78	82.8	82.9
12	5.63	5.62	5.62	5.58	5.57	126.2	126.3
11	6.65	6.67	6.67	6.67	6.64	131.6	131.8
10	6.00	6.00	6.01	6.01	5.99	127.3	127.3
9	5.55	5.54	5.55	5.54	5.54	135.4	135.2
OOH	9.50	9.52	9.38	9.08	9.04		

^a^ Ref [35]; ^b^ Ref [4].

**Table 3:** ^1^H-NMR quantification data of hydroperoxides and *endo*-hydroperoxides produced during the oxidation of methyl linolenate ^a^.

Hydroperoxide *δ* (^1^H), ppm	Integration Data (%)	Integration Data (%) of Total Hydroperoxides	Assignment
7.92	5.78%	29% (33.0%) ^b^	*10-Trans, 12-cis, 15-cis*-9-OOH
8.04	2.89%	14% (10.8%) ^b^	*9-Cis, 13-trans, 15-cis*-12-OOH
8.05	8.23%	41% (43.9%) ^b^	*10-Cis, 13-cis, 15-trans*-16-OOH
8.06	3.08%	15% (12.3%) ^b^	*9-Cis, 11-trans, 15-cis*-13-OOH
9.08	1.80%		9-*Cis*, 11-*trans*, *syn erythro*, 16-OOH *endo*-hydroperoxide
9.12	1.36%		13-T*rans*, 15-*cis*, *syn erythro*, 9-OOH *endo*-hydroperoxide
9.50	2.40%		9-*Cis*, 11-*trans*, *syn threo*, 16-OOH *endo*-hydroperoxide
9.55	1.88% ^c^		13-*Trans*, 15-*cis*, *syn threo*, 9-OOH *endo*-hydroperoxide^c^

^a^ Heated at 40 °C for 48 h of the present work. ^b^ % Integration data from the literature at 40 °C [36]. ^c^ The concentration of the *threo endo*-hydroperoxide was estimated to be 1.68 mM in the NMR tube, which can be compared with the detection limit of 3 μM of the 800 MHz NMR instrument.

## Supplementary Materials

The following are available online, Figure S1: Selected ^1^H-NMR chemical shift ranges of the endo-hydroperoxide (OOH) region (A), and CH-OOH region (B), Figure S2: Proposed mechanism of the formation of two diastereomeric pairs of 9-cis, 11-trans-16-OOH, and 13-trans, 15-cis-9-OOH linolenate endo-hydroperoxides, Figure S3: Minimum energy structures of the two pairs of diastereomers of 9-cis, 11-trans-16-OOH endo-hydroperoxides with a discrete solvation molecule of CHCl_3_ in IEFPCM (CHCl_3_), Figure S4: Power attenuation details of the soft Gaussian 180o selective refocusing pulse of the selmlgp pulse program, Table S1: Critical hydroperoxide OOH and CH-OOH ^1^H-NMR chemical shifts for the identification of hydroperoxides and endo-hydroperoxides of methyl linolenate, Table S2: Comparison of computational [B3LYP/6-311G+d (2d, p)] ^1^H-NMR chemical shifts of the two pairs of diastereomeric 16-OOH endo-hydroperoxide models, with energy minimization at the APFD/6-31+G(d) level, with the experimental chemical shifts of the full length molecules, Table S3: Comparison of computational [B3LYP/6-311G+d (2d, p)] ^1^H-NMR chemical shifts of the 10-cis, 13-cis, 15-trans-16-OOH hydroperoxide model, with energy minimization at the APFD/6-31+G(d) level, with the experimental chemical shifts of the full length molecule, Table S4: Comparison of experimental and computational ^1^H-NMR chemical shifts of the full length diastereomeric 9-cis, 11-trans-l6-OOH endo-hydroperoxides with energy minimization at the APFD/6-31+G(d):PM6 level, Table S5: Conformational and structural properties of the full length diastereomeric 9-cis, 11-trans-16-OOH endo-hydroperoxides with energy minimization using the APFD/6-31+G(d):PM6 method, Table S6: ^1^H-NMR chemical shifts of hydroperoxides of methyl linolenate.
