# Supplementary material for: NMR and Computational Studies as Analytical and High-Resolution Structural Tool for Complex Hydroperoxides and Diastereomeric Endo-Hydroperoxides of Fatty Acids in Solution-Exemplified by Methyl Linolenate
Source: Molecules. 2020 Oct 23;25(21):4902. doi: 10.3390/molecules25214902 (PMC7660186; doi:10.3390/molecules25214902)
Supplement: Supplementary file 1 [file molecules-25-04902-s001.pdf]

NMR and Computational Studies as Analytical and High Resolution Structural Tool for Complex Hydroperoxides and Diastereomeric *Endo*-hydroperoxides of Fatty Acids in Solution - Exemplified by Methyl Linolenate

Raheel Ahmed[1], Panayiotis C. Varras[2], Michael G. Siskos[2], Hina Siddiqui[1*], M. Iqbal Choudhary[1,3], and Ioannis P. Gerothanassis[1,2*]

[1]H.E.J. Research Institute of Chemistry, International Center for Chemical and Biological Sciences, University of Karachi 75270, Pakistan; E-mails: hinahej@gmail.com, genius88raheel@gmail.com, iqbal.choudhary@iccs.edu

[2]Section of Organic Chemistry and Biochemistry, Department of Chemistry, University of Ioannina, GR-45110, Greece; E-mail: igeroth@uoi.gr, msiskos@uoi.gr, panostch@gmail.com

[3]Department of Biochemistry, Faculty of Science, King Abdulaziz University, Jeddah 214412, Saudi Arabia; E-mail: iqbal.choudhary@iccs.edu

* Correspondence: hinahej@gmail.com (H.S.); igeroth@uoi.gr (I.P.G.)

SUPPLEMENTARY MATERIAL

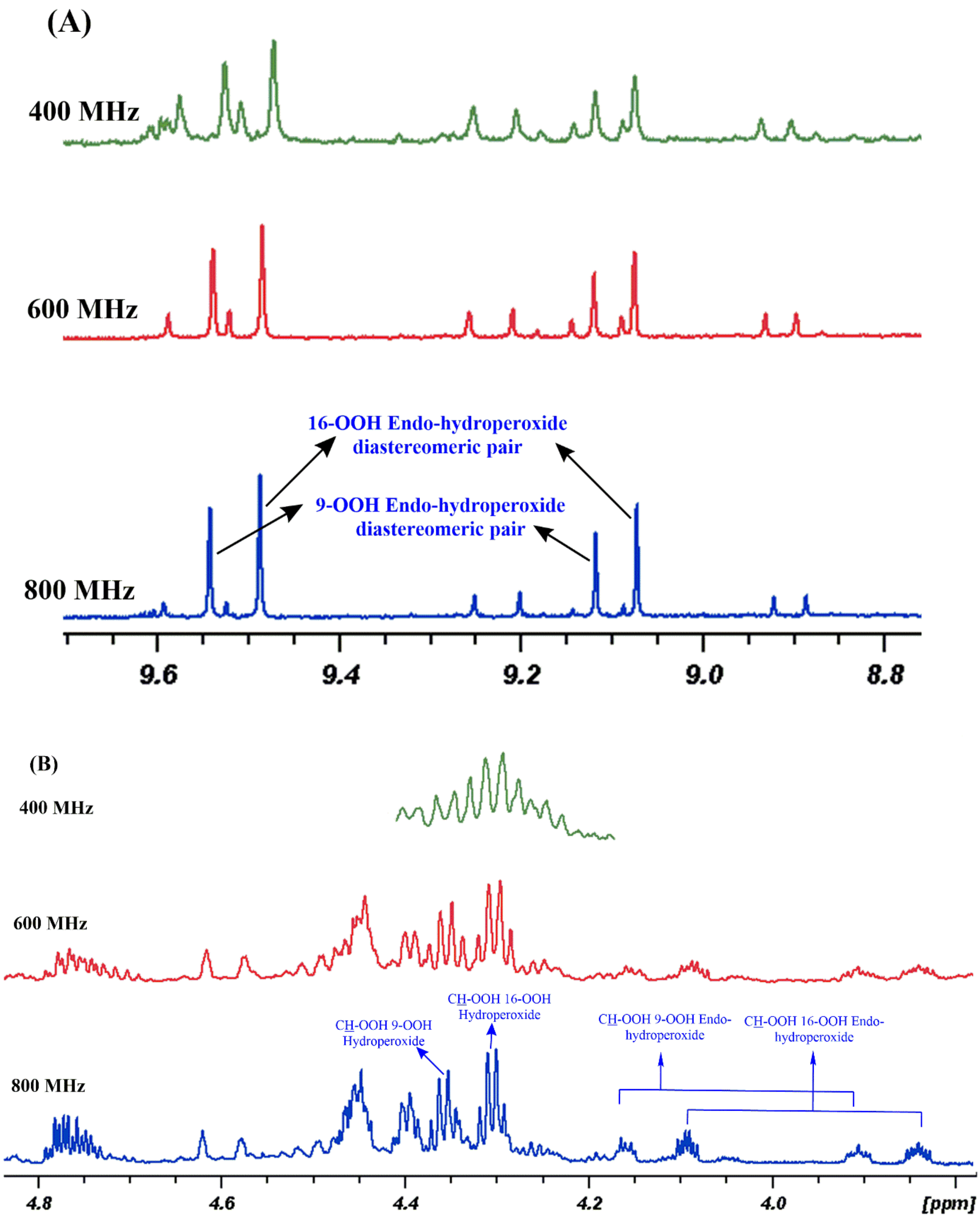

Figure S1. Selected ^{1}H-NMR chemical shift ranges of the *endo*-hydroperoxide (OOH) region (A), and C<u>H</u>-OOH region (B), using 800 MHz (number of scans = 8), 600 MHz (number of scans = 8), and 400 MHz (number of scans = 128) instruments.

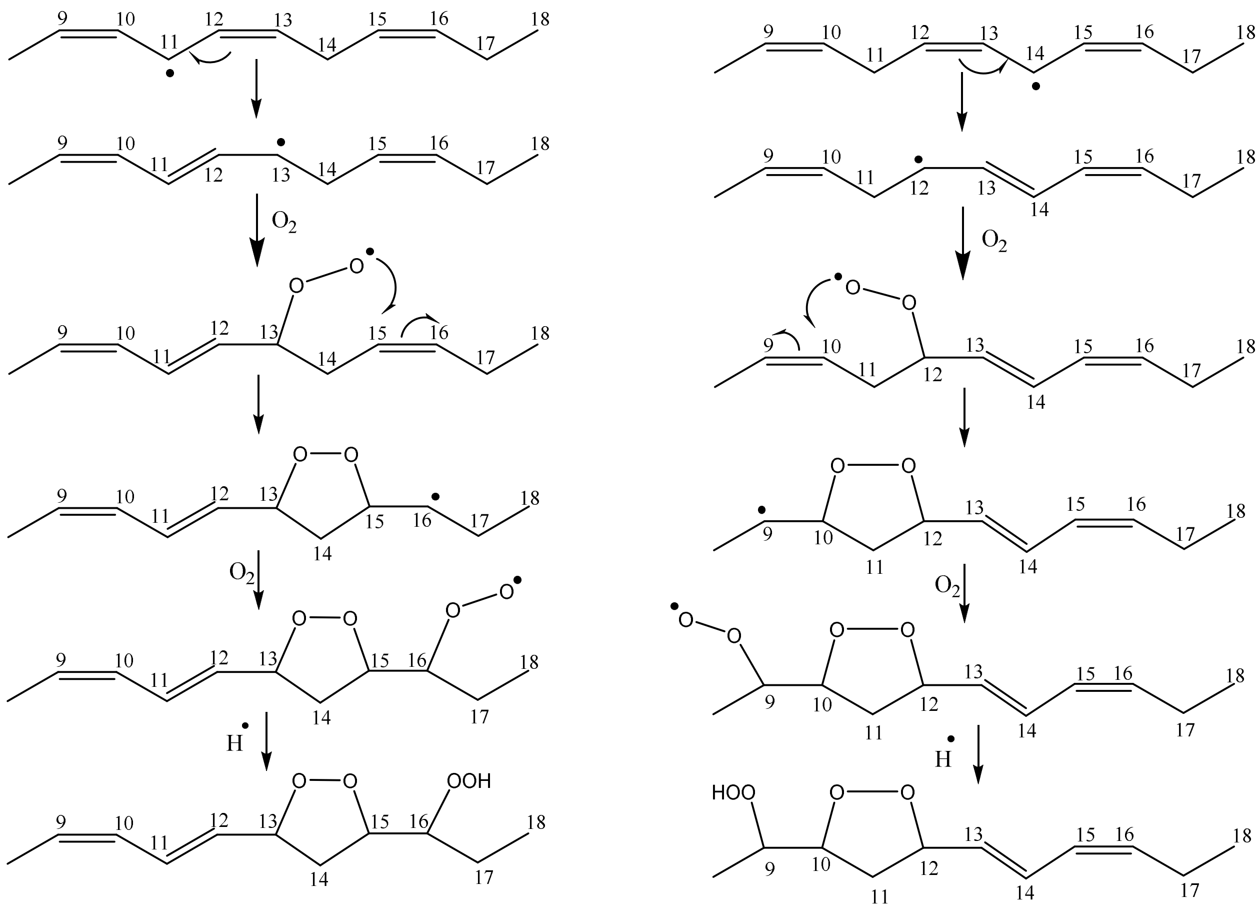

Figure S2. Proposed mechanism of the formation of two diastereomeric pairs of 9-*cis*, 11-*trans*-16-OOH, and 13-*trans*, 15-*cis*-9-OOH linolenate *endo*-hydroperoxides.

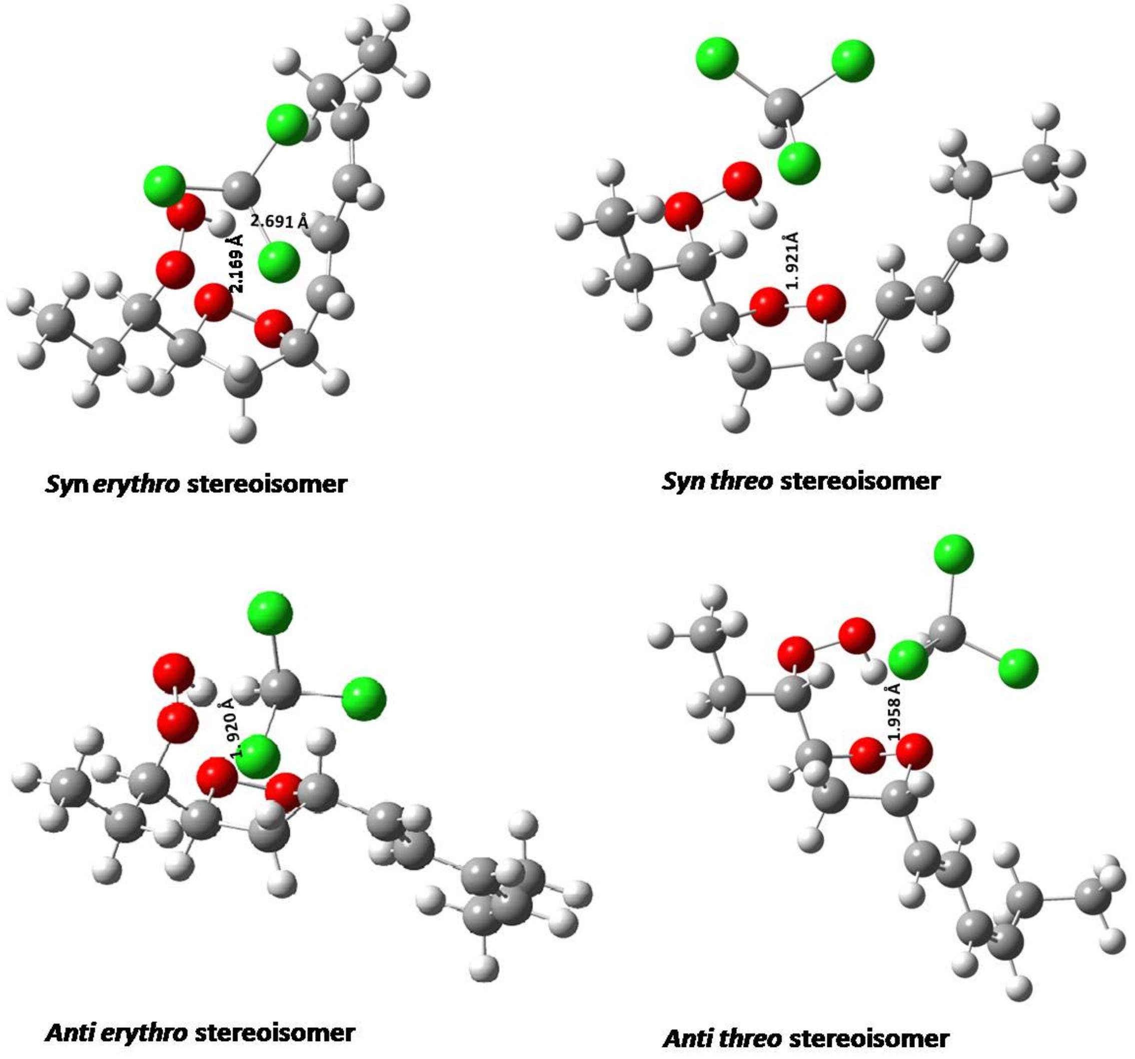

Figure S3. Minimum energy structures of the two pairs of diastereomers of 9-*cis*, 11-*trans*-16-OOH *endo*-hydroperoxides with a discrete solvation molecule of $CHCl_3$ in IEFPCM ($CHCl_3$).

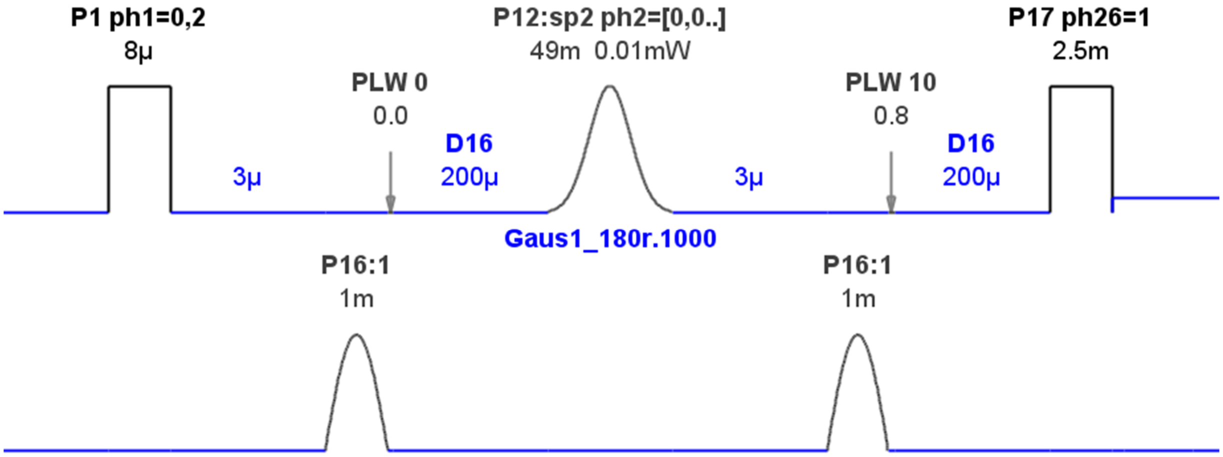

Figure S4. Power attenuation details of the soft Gaussian 180º selective refocusing pulse of the selmlgp pulse program.

Table S1. Critical hydroperoxide OOH and C<u>H</u>-OOH ^{1}H-NMR chemical shifts for the identification of hydroperoxides and *endo*-hydroperoxides of methyl linolenate.

**Hydroperoxide δ (^{1}H), ppm**	**C<u>H</u>-OOH δ (^{1}H), ppm**	**Assignment**
7.92	4.39	*10-Trans, 12-cis, 15-cis*-9-OOH
8.05	4.34	*10-Cis, 13-cis, 15-trans*-16-OOH
9.08	3.87	9-*Cis*, 11-*trans*, *syn erythro*, 16-OOH *endo*-hydroperoxide
9.12	3.94	13-*Trans*, 15-*cis*, *syn erythro*, 9-OOH *endo*-hydroperoxide
9.50	4.13	9-*Cis*, 11-*trans*, *syn threo*, 16-OOH *endo*-hydroperoxide
9.55	4.19	13-*Trans*, 15-*cis*, *syn threo*, 9-OOH *endo*-hydroperoxide

Table S2. Comparison of computational [B3LYP/6-311G+d (2d, p)] ^{1}H-NMR chemical shifts of the two pairs of diastereomeric 16-OOH *endo*-hydroperoxide models, with energy minimization at the APFD/6-31+G(d) level, with the experimental chemical shifts of the full length molecules: **a.** with PCM, **b.** with one discrete solvation molecule of $CHCl_3$ in PCM.

			GIAO δ(^{1}H), ppm				*CSGT* δ(^{1}H), ppm			
Threo	Proton No	Experimental δ(^{1}H), ppm	SYN		ANTI		SYN		ANTI	
			**a**	**b**	**a**	**b**	**a**	**b**	**a**	**b**
	-OOH	9.50	9.97	10.52	10.28	10.23	9.47	10.03	9.89	9.69
	18	1.05	1.04	1.16	1.09	1.13	0.91	0.90	0.97	0.96
	17(a)	1.49	1.24	1.36	1.27	1.29	1.19	1.17	1.17	1.18
	17(b)		1.03	1.11	1.06	1.11	0.93	0.94	0.96	0.98
	16	4.13	4.30	4.39	3.86	4.20	4.06	3.92	3.59	3.79
	15	4.49	4.13	4.28	4.18	3.28	3.87	3.98	3.91	3.93
	14(a)	2.84	2.88	2.89	2.48	2.52	2.64	2.62	2.23	2.30
	14(b)	2.43	2.11	1.88	2.42	2.46	1.95	1.65	2.24	2.45
	13	4.81	5.04	5.05	5.11	5.44	4.76	4.68	4.80	4.84
	12	5.63	5.98	5.88	5.65	5.62	5.66	5.66	5.42	5.39
	11	6.67	7.51	7.11	7.29	7.25	7.16	6.66	6.84	6.82
	10	6.00	6.31	6.34	6.39	6.34	6.00	5.88	5.98	5.97
	9	5.55	5.93	5.81	5.84	5.87	5.64	5.51	5.60	5.58
	8	2.18	2.45	2.45	2.43	2.42	2.32	1.88	2.27	2.26
Erythro	-OOH	9.08	8.61	8.93	9.50	10.09	8.28	8.47	9.21	9.73
	18	1.07	1.06	1.13	1.11	1.14	0.95	0.93	0.99	0.97
	17(a)	1.66	1.35	1.43	1.37	1.55	1.27	1.26	1.28	1.30
	17(b)	1.57	1.10	1.16	1.15	1.47	1.02	1.03	1.04	1.29
	16	3.87	4.08	4.21	4.25	4.33	3.86	3.94	4.02	4.01
	15	4.49	4.37	4.45	4.61	4.73	4.08	4.12	4.34	4.39
	14(a)	2.88	2.68	2.78	2.73	2.82	2.42	2.43	2.50	1.80
	14(b)	2.23	2.30	2.44	2.05	2.14	2.16	2.15	1.80	2.40
	13	4.81	5.09	5.16	5.94	5.26	4.74	4.75	4.67	4.75
	12	5.58	5.99	5.99	6.13	6.11	5.70	5.76	5.83	5.69
	11	6.65	7.39	7.35	6.96	6.89	7.29	6.77	6.60	6.48
	10	6.00	6.37	6.71	6.18	6.23	6.03	6.03	5.96	5.90
	9	5.54	5.96	6.26	5.86	5.84	5.66	5.60	5.58	5.56
	8	2.18	2.36	2.36	2.46	2.54	2.20	2.14	2.21	2.19

Table S3. Comparison of computational [B3LYP/6-311G+d (2d, p)] ^{1}H-NMR chemical shifts of the 10-*cis,* 13-*cis,* 15-*trans*-16-OOH hydroperoxide model, with energy minimization at the APFD/6-31+G(d) level, with the experimental chemical shifts of the full length molecule.

Proton no	Experimental δ (^{1}H), ppm	Calculated δ(^{1}H), ppm^a	Calculated δ(^{1}H), ppm^b
18	0.95	0.88	1.00
17(a)	1.72	2.59	2.62
17(b)	1.55	1.30	1.47
16	4.34	4.26	4.74
15	5.62	5.53	5.66
14	6.62	7.38	7.40
13	6.03	6.28	6.33
12	5.47	5.79	5.85
11	2.98	3.15	3.09
10	5.42	5.89	6.03
9	5.36	5.88	6.09
-OOH	8.06	6.71	7.23

^{a} With PCM; ^{b} with one discrete solvation molecule of chloroform in PCM.

Table S4. Comparison of experimental and computational ^{1}H-NMR chemical shifts of the full length diastereomeric 9-*cis,* 11-*trans*-l6-OOH *endo*-hydroperoxides with energy minimization at the APFD/6-31+G(d):PM6 level: **a.** in PCM, **b.** with one solvation molecule of chloroform in PCM.

	**Proton No**	**Experimental δ(^{1}H), ppm**	**GIAO δ(^{1}H), ppm**		**CSGT δ(^{1}H), ppm**	
Syn threo			**a**	**b**	**a**	**b**
	-OOH	9.50	9.93	10.51	9.46	10.04
	18	1.05	1.04	1.14	0.91	0.90
	17(a)	1.49	1.19	1.22	1.07	1.06
	17(b)		1.19	1.08	1.07	0.95
	16	4.13	4.34	4.37	4.01	3.94
	15	4.49	4.17	4.30	3.82	3.99
	14(a)	2.84	2.89	2.99	2.64	2.62
	14(b)	2.43	2.17	1.88	1.92	1.66
	13	4.81	5.10	5.04	4.74	4.68
	12	5.63	6.05	5.91	5.71	5.67
	11	6.67	7.57	7.14	7.15	6.71
	10	6.00	6.30	6.27	6.00	5.92
	9	5.55	6.27	6.17	5.91	5.84
	8	2.18	2.16	2.50	2.47	2.05
	7		1.73	1.68	2.02	1.39
	6		1.78	1.61	2.13	1.54
	5		1.70	1.47	2.03	1.42
	4		1.54	1.52	1.91	1.40
	3		1.90	2.08	2.40	1.92
	2		2.88	2.97	3.08	2.68
	OCH_3		3.82	3.86	4.06	3.66
Syn erythro	-OOH	9.08	8.68	8.99	8.30	8.49
	18	1.07	1.09	1.15	0.97	0.94
	17(a)	1.66	1.38	1.43	1.28	1.27
	17(b)	1.57	1.14	1.20	1.03	1.04
	16	3.87	4.11	4.24	3.87	3.94
	15	4.49	4.38	4.46	4.10	4.12

14(a)	2.88	2.71	2.76	2.42	2.43
14(b)	2.23	2.35	2.49	2.17	2.15
13	4.81	5.10	5.15	4.75	4.75
12	5.58	5.99	6.23	5.71	5.75
11	6.65	7.40	7.31	6.90	6.80
10	6.00	6.45	6.61	6.05	6.02
9	5.54	6.25	6.06	5.97	5.66
8	2.18	2.33	2.31	2.54	2.05
7		1.53	1.48	1.67	1.35
6		1.60	1.64	1.70	1.52
5		1.50	1.59	1.54	1.49
4		1.45	1.53	1.60	1.48
3		1.77	1.90	1.92	1.77
2		2.66	2.89	2.84	2.68
OCH_3		3.82	3.81	3.64	3.63

Table S5. Conformational and structural properties of the full length diastereomeric 9-*cis,* 11-*trans*-16-OOH *endo*-hydroperoxides with energy minimization using the APFD/6-31+G(d):PM6 method.

Optimization	C–O (Å)	O–O (Å)	O–H (Å)	C(16) – O – O – H	C(17) – C(16) – O – O	C(15) – C(16) – O – O	(O)H----O (Å)	O(H) ----O (Å)	O–H…O
Syn threo [a]	1.427	1.433	0.978	74.2°	157.6°	-81.9°	1.991	2.743	131.9°
Syn threo [b]	1.429	1.433	0.980	68.9°	158.6°	-80.4°	1.920	2.469	135.4°
Syn erythro [a]	1.426	1.430	0.979	-88.5°	-155.8°	82.7°	2.397	2.858	132.3°
Syn erythro [b]	1.426	1.431	0.979	-78.9°	-154.3°	83.8°	2.169	3.039	119.0°

[a] IEF-PCM-$CHCl_3$; [β] with one solvation molecule of chloroform (IEF-PCM-$CHCl_3$)

Table S6. ^{1}H-NMR chemical shifts of hydroperoxides of methyl linolenate.

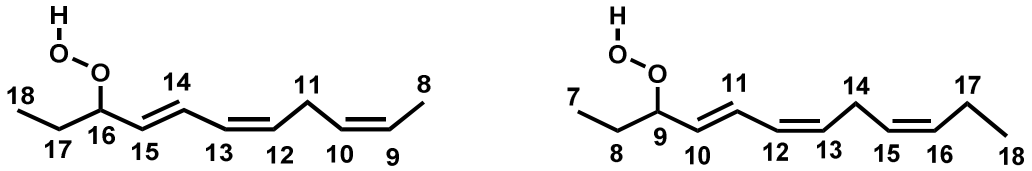

	**9-*cis,* 12-*cis,* 14-*trans*-16-OOH hydroperoxide**	**10-*trans,* 12-*cis,* 15-*cis*-9-OOH hydroperoxide**
**Proton no.**	***δ*** **(^{1}H), ppm**	***δ*** **(^{1}H), ppm**
18	0.95	
17(a)	1.72	
17(b)	1.55	
16	4.34	5.33
15	5.62	5.43
14	6.62	2.96
13	6.03	5.48
12	5.47	6.03
11	2.95	6.61
10	5.42	5.61
9	5.36	4.39
8(a)		1.48
8(b)		1.66
OOH	8.06	7.93
